# *In-situ* coupling between kinase activities and protein dynamics within single focal adhesions

**DOI:** 10.1038/srep29377

**Published:** 2016-07-07

**Authors:** Yiqian Wu, Kaiwen Zhang, Jihye Seong, Jason Fan, Shu Chien, Yingxiao Wang, Shaoying Lu

**Affiliations:** 1 Department of Bioengineering, University of California, San Diego, La Jolla, CA 92093, USA; 2 Center for Neuro-Medicine, Brain Science Institute, Korea Institute of Science and Technology (KIST), Seoul, South Korea; 3 Institute of Engineering in Medicine, University of California, San Diego, La Jolla, CA 92093, USA; 4 Center of Computational Mathematics, University of California, San Diego, La Jolla, CA 92093, USA

## Abstract

The dynamic activation of oncogenic kinases and regulation of focal adhesions (FAs) are crucial molecular events modulating cell adhesion in cancer metastasis. However, it remains unclear how these events are temporally coordinated at single FA sites. Therefore, we targeted fluorescence resonance energy transfer (FRET)-based biosensors toward subcellular FAs to report local molecular events during cancer cell adhesion. Employing single FA tracking and cross-correlation analysis, we quantified the dynamic coupling characteristics between biochemical kinase activities and structural FA within single FAs. We show that kinase activations and FA assembly are strongly and sequentially correlated, with the concurrent FA assembly and Src activation leading focal adhesion kinase (FAK) activation by 42.6 ± 12.6 sec. Strikingly, the temporal coupling between kinase activation and individual FA assembly reflects the fate of FAs at later stages. The FAs with a tight coupling tend to grow and mature, while the less coupled FAs likely disassemble. During FA disassembly, however, kinase activations lead the disassembly, with FAK being activated earlier than Src. Therefore, by integrating subcellularly targeted FRET biosensors and computational analysis, our study reveals intricate interplays between Src and FAK in regulating the dynamic life of single FAs in cancer cells.

Focal adhesions (FAs) are prominent intracellular molecular complexes containing hundreds of residential proteins, including integrin receptors, tyrosine kinases, and scaffolding proteins^1^,^2^,^3^. Located at the interface of intracellular actin cytoskeleton and extracellular matrix, FAs undergo dynamic modifications involving their assembly, maturation, turnover, and disassembly^1^,^4^. FA proteins have been shown to form 3D nano-structures and move as slippery clutches in cells^5^,^6^. Increasing evidence indicates that FA dynamics is critically involved in important cellular processes, such as cancer metastasis, stem cell differentiation, and immune response, as well as biochemical and mechano-sensing of the surrounding microenvironment^7^,^8^,^9^,^10^,^11^. While the dynamic regulation of FA proteins and their structural segregation have been under intense investigation, it remains unclear how molecular activities are coupled to the protein localization in guiding the FA assembly and disassembly dynamics at individual single FA sites.

The process of cellular adhesion, involves the dynamic regulation of FAs, with rapid assembly and turnover of FA complexes^12^,^13^. At the start of cell adhesion, integrin receptors at the cellular surface bind to matrix proteins, causing integrin activation and clustering to recruit adapter proteins and tyrosine kinases, such as paxillin, talin, Src family kinase (SFK) and focal adhesion kinase (FAK), to the initial FAs^14^. These kinases can phosphorylate local substrate molecules and create docking sites for the recruitment and assembly of FA complexes^15^,^16^. The activation of tyrosine kinases coordinates with the transient increase of membrane tension to regulate FA traction force and FA turnover, as well as promote sustained cell spreading^17^,^18^,^19^,^20^,^21^. The complex roles of tyrosine kinases reflect a sophisticated and dynamic regulation mechanism in single FAs. To elucidate the dynamic roles of kinases in regulating FA kinetics, there is a great need for new imaging tools that allow the monitoring and analysis of multiple molecular events in single FAs.

Genetically encoded fluorescence resonance energy transfer (FRET) biosensors have been widely used to visualize subcellular molecular activities at single-cell level^22^,^23^. We have previously developed FAK and Src FRET biosensors and demonstrated their capabilities of monitoring the corresponding kinase activities with high sensitivity and specificity^24^,^25^. Utilizing these biosensors, we demonstrated that at the plasma membrane, most of the Src activation occurs outside the lipid-rafts microdomain^26^, whereas FAK is mainly activated within the lipid-rafts microdomain^25^. Here we have engineered novel FA-targeting (FAT) biosensors with the C-terminal FAT region of FAK, to monitor and track kinase activities at individual FA sites^27^,^28^,^29^,^30^. Therefore, these FAT-Src and FAT-FAK biosensors should allow the visualization and quantification of the local Src and FAK activities, respectively, with single-FA precision.

While subcellularly targeted FRET biosensors enable the visualization of molecular signals with high resolution, computational analysis is indispensable for integrating multiple signals and deciphering regulatory parameters from live-cell images^18^,^23^,^31^,^32^. To elucidate the dynamic coupling between the biochemical kinase activities and structural FA dynamics we integrated the new FA-targeting biosensors, single-FA tracking, and cross-correlation analysis methods to quantitatively evaluate the temporal coordination of these molecular events at the single-FA level. Our analysis focused on elucidating the governing mechanism and temporal orders among the biochemical kinase activities and biophysical FA dynamics in individual FAs during the adhesion of cancer cells. Such fundamental insights cannot be gained via traditional methods, because biochemical assays that average signals from a large number of cells and FAs cannot reveal the precise temporal coordination of molecular activations within single FA sites, especially if different FAs undergo non-synchronized regulations.

## Results

### FAT-FRET biosensors for monitoring Src and FAK kinase activities at single FA sites

**Figure 1:**
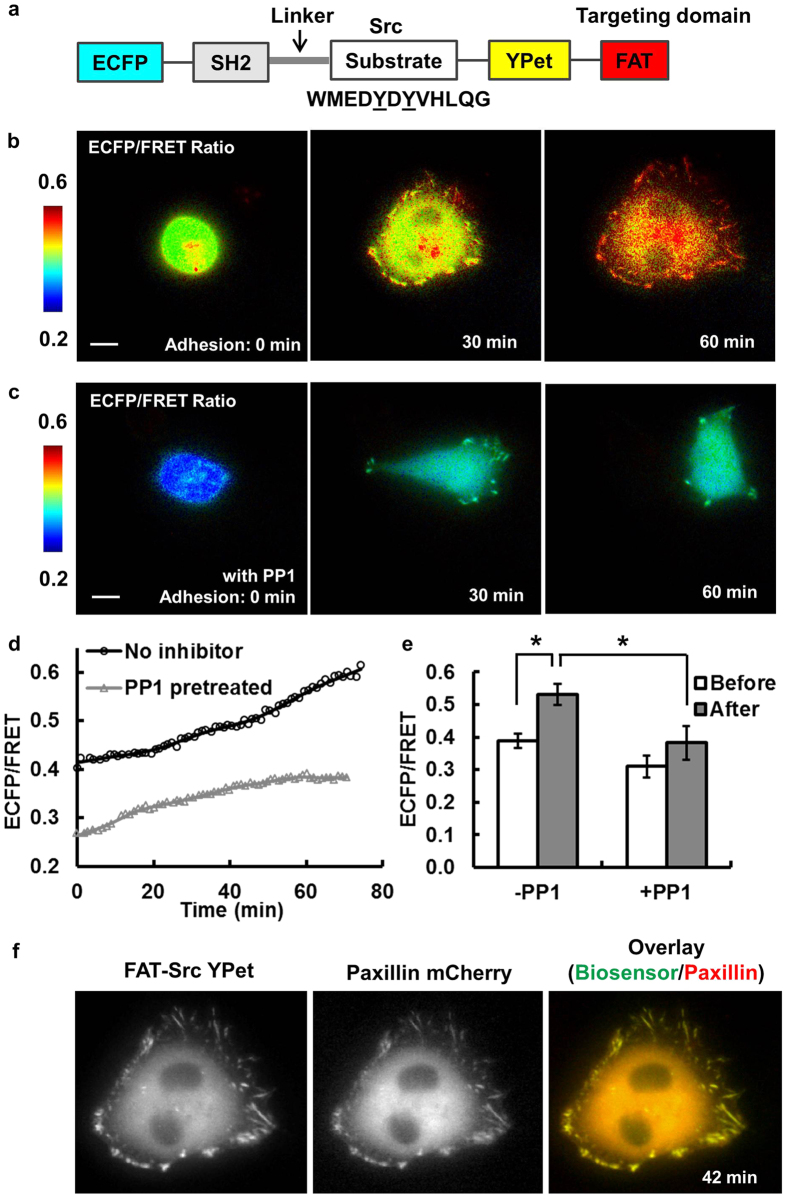
Engineer and characterize the focal-adhesion-targeting FAT-Src FRET biosensor. **(a**) The schematics of the FA-targeting Src FRET biosensor. **(b)** The ECFP/FRET ratio images of a representative cell during the adhesion process. **(c)** The ECFP/FRET ratio images of a control cell pretreated with Src inhibitor PP1. **(d)** The time courses of the ECFP/FRET ratio in the cell shown in (**b**) (black line with circles) and that in (**c**) (gray line with triangles). **(e)** The average ECFP/FRET ratio values before (0–3 min, white) and after (33–36 min, gray) the appearance of FAs in cells with (*N1* = *8*) or without PP1 treatment (*N2* = *5*). * indicates significant difference by Bonferroni multiple comparison test *(p* < *0.05)*. **(f)** The YPet intensity (left) and mCherry image (middle) of a cell co-transfected with the FAT-Src biosensor and mCherry paxillin, with the overlaid image shown on the right. Scale bar: 10 μm.

**Figure 2:**
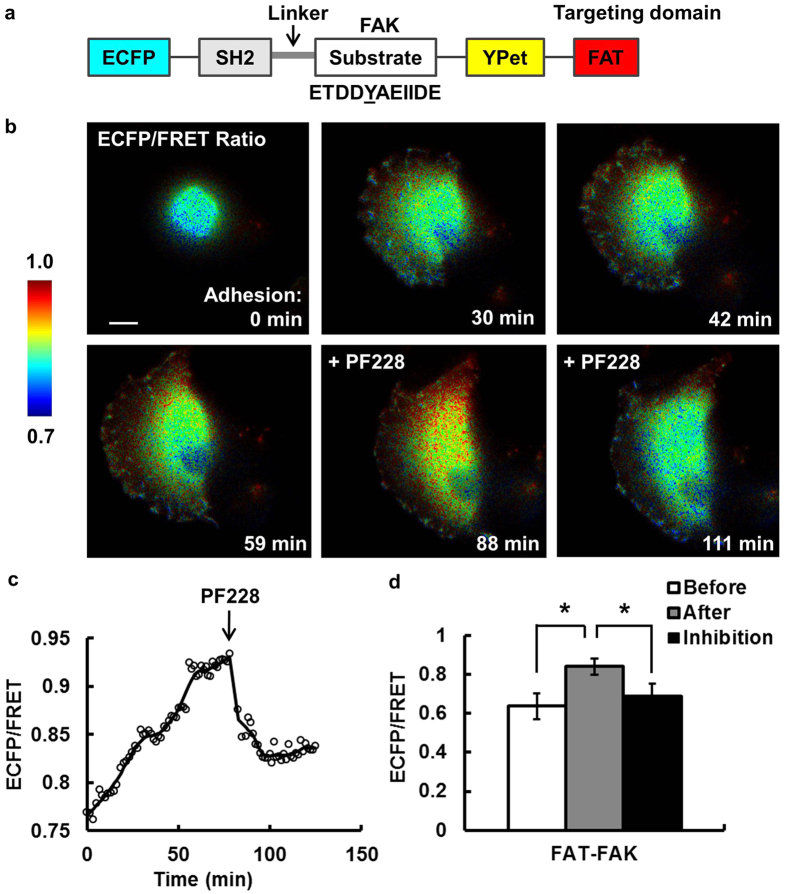
The FAT-FAK FRET biosensor is sensitive and specific in detecting FAK activity in live cells. **(a)** The schematics of the FAT-FAK FRET biosensor. **(b)** The ECFP/FRET ratio images of a representative cell before and after adhesion, and that after FAK inhibition. **(c)** The time course of the average ECFP/FRET ratio in the cell shown in (**b**). **(d)** The average ECFP/FRET ratio values before (0–4 min, white) and after (75–78 min, gray) the appearance of FAs, and that after inhibitor treatment (~105–108 min, 0.69 ± 0.067, black). * indicates significant difference from the other groups by a paired Bonferroni multiple comparison test (*p* < *0.05*, *N* = *3*). Scale bar: 10 μm.

To monitor local kinase activities with high spatiotemporal resolution, we engineered FRET-based Src and FAK biosensors to target to FAs, namely the FAT-Src and FAT-FAK biosensors (Figs 1 and 2). The FAT-Src biosensor contains sequentially a Src SH2 domain, a flexible linker, and a Src specific substrate sequence, which are concatenated in between the enhanced cyan fluorescent protein (ECFP) and yellow fluorescent protein (YPet) FRET pair, with YPet connected to the FAT domain for FA localization (Fig. 1a). Active Src kinase can cause the tyrosine phosphorylation of the substrate and the subsequent increase of ECFP/FRET emission ratio^24^,^26^,^33^. Therefore, the ECFP/FRET ratio can be used to represent subcellular Src kinase activity. Herein, we applied the FA-localized FAT-Src biosensor for reporting the Src activities within each individual FA site during cell adhesion.

MDA-MB-231 breast cancer cells expressing FAT-Src biosensors were seeded onto fibronectin-coated cover glass to allow cell adhesion and spreading (Fig. 1b and Supplementary Video S1). As shown in a representative cancer cell (Fig. 1b,d), the intracellular ECFP/FRET ratio increased ~10% and ~40% of basal level at 30 min and 60 min after initial adhesion, respectively. Quantified results showed an average increase of ~40% in ECFP/FRET ratio across multiple cells, clearly representing a significant Src activation during cell adhesion (Fig. 1e). The intensity profile of the FAT-Src biosensor showed a clear FA localization in clusters at the cell periphery, although diffusive fluorescence signals were seen at perinuclear structures, possibly reflecting that some biosensor cargoes were trapped during the transportation process after protein synthesis (Fig. 1b). These results indicate that FAT-Src biosensors correctly localize to FAs and can be used to visualize Src activation within these individual structures with high sensitivity during cell adhesion.

To confirm the localization of the FAT-Src biosensor, the YPet intensity pattern of the biosensor was compared with that of the FA protein, mCherry-paxillin. As shown in Fig. 1f and Supplementary Fig. S1a,b, the YPet signals overlapped with mCherry-paxillin throughout the adhesion process at cell periphery. Slightly more paxillin was observed at perinuclear regions than biosensor signals, probably representing diffusive background of unprocessed fusion proteins trapped in organelles such as Golgi complex and ER (Fig. 1f). Correlation analysis showed a high time-average Pearson coefficient of 0.986 ± 7e–4 between the two images (Fig. S1). This co-localization was observed in all four cells studied with FAT-Src biosensors and mCherry-paxillin. Therefore, our results indicate that the FAT-Src biosensors are recruited to peripheral FAs and undergo turnover with similar spatiotemporal dynamics as the FA protein paxillin.

Both the original FAK and Src biosensors have been thoroughly examined and characterized to demonstrate a high specificity toward their desired target kinases^34^,^35^. The addition of fusion tag FAT is expected to enhance the subcellular efficiency without altering the specificity of these biosensors. Nevertheless, to further characterize the specificity of our FAT-Src biosensor, cells expressing the biosensors were treated with the SFK inhibitor PP1 and monitored during the adhesion process. PP1 pretreatment (10 μM for 1 hr) significantly blocked the adhesion process, as ~50% of the cells did not attach, indicating the importance of SFK kinase activity in adhesion. In the group of PP1-treated cells that did adhere, the quantified Src ECFP/FRET ratio showed significantly reduced values (Fig. 1c–e and Supplementary Video S2). The cells treated with PP1 also had less expansion with a reduced number of FAs than those without PP1 (Fig. 1c–d and Supplementary Video S2). Taken together, these results indicate that our FAT-Src biosensors can specifically detect dynamic Src kinase activation in single FAs, which is important for cancer cell adhesion and expansion.

Using a similar strategy, we engineered a FAT-FAK biosensor^34^ (Fig. 2a). The FAT-FAK biosensor allowed the visualization of FAK activation with high sensitivity during the adhesion process of MDA-MB-231 cells (Fig. 2b and Supplementary Video S3). Again, the majority of FAT-FAK biosensors localized correctly to FAs, with some appeared at perinuclear regions. A representative cell showed ~22% increase of ECFP/FRET ratio during adhesion (Fig. 2c). Pretreatment of the cells with PF228 (FAK inhibitor, 1 μM for 1 hr) blocked cell adhesion, while the addition of PF228 (1 μM) during imaging caused a significant decrease in the ECFP/FRET ratio of the biosensors, confirming the specificity of the FRET signals (Fig. 2c,d). These results demonstrate that our FAT-FAK biosensors can be used to monitor FAK activation at single FAs during the adhesion of cancer cells with high sensitivity and specificity.

In a cell expressing both the FAT-FAK biosensor and mCherry-paxillin, YPet signals overlapped precisely with mCherry patterns with a time-averaged Pearson coefficient of 0.994 ± 9e–5 during adhesion (Supplementary Fig. S1c), indicating that the FAT-FAK biosensor and paxillin were highly co-localized. Quantitative plots confirmed similar dynamics between FAT-FAK-YPet and mCherry-paxillin (Supplementary Fig. S1d). This co-localization was observed in all five cells co-expressing FAT-FAK biosensors and mCherry-paxillin. Therefore, the YPet signal of the FAT-FAK biosensor can be used to assess and quantify the dynamic recruitment and turnover of the FA structure highlighted by paxillin^36^,^37^. In addition, YPet images were collected with YPet excitation and YPet emission, which is different and independent of FRET images collected by ECFP excitation and YPet emission. As a result, a single transfection of a FAT-biosensor can allow the simultaneous tracking and quantification of the biochemical kinase activity (via ECFP/FRET ratio) and the biophysical FA dynamics (via YPet intensity).

To examine the general applicability of our FA-tagged FRET biosensors, we transfected the biosensors into a different cell line, mouse embryonic fibroblasts (MEFs), and monitored the adhesion process. Similar to that in the MDB-MD-231 cells, the FAT-Src biosensors in MEF cells localized to FAs, with the ECFP/FRET ratio increased ~50% in 30–60 minute after adhesion (Supplementary Fig. S2). Meanwhile, MEF cells expressing the FAT-FAK biosensors also showed correct FA-localization and ~30% increase in the ECFP/FRET ratio after adhesion (Supplementary Fig. S2). Altogether, these results indicate that our FA-targeted FRET biosensors provide new tools to visualize and quantitatively analyze kinase activities in single FAs and that the FA-target strategy has general utility in engineering FRET biosensors for monitoring molecular activities with subcellular resolution.

### Quantification of kinase activities and FA dynamics in single FAs by feature detection and single-FA tracking during adhesion of cancer cells

During the adhesion process, cells undergo dramatic expansion and FAs are highly dynamic. The fluorescence intensity images of the MDA-MB-231 breast cancer cells were significantly smaller and brighter at the beginning of adhesion than those after adhesion, with ~10-fold increase of the cell area (Figs 1 and 2). In general, there were no visible FAs initially. When the FAs became detectable in the YPet intensity images, the number of FAs quickly increased to 20 or more per cell within 30 min (Figs 1 and 2). Meanwhile, cells expanded before they started migration. Such drastic changes in cell shape and FA dynamics require accurate detection and tracking methods to precisely quantify the local kinase activities and FA dynamics at single FA sites.

**Figure 3:**
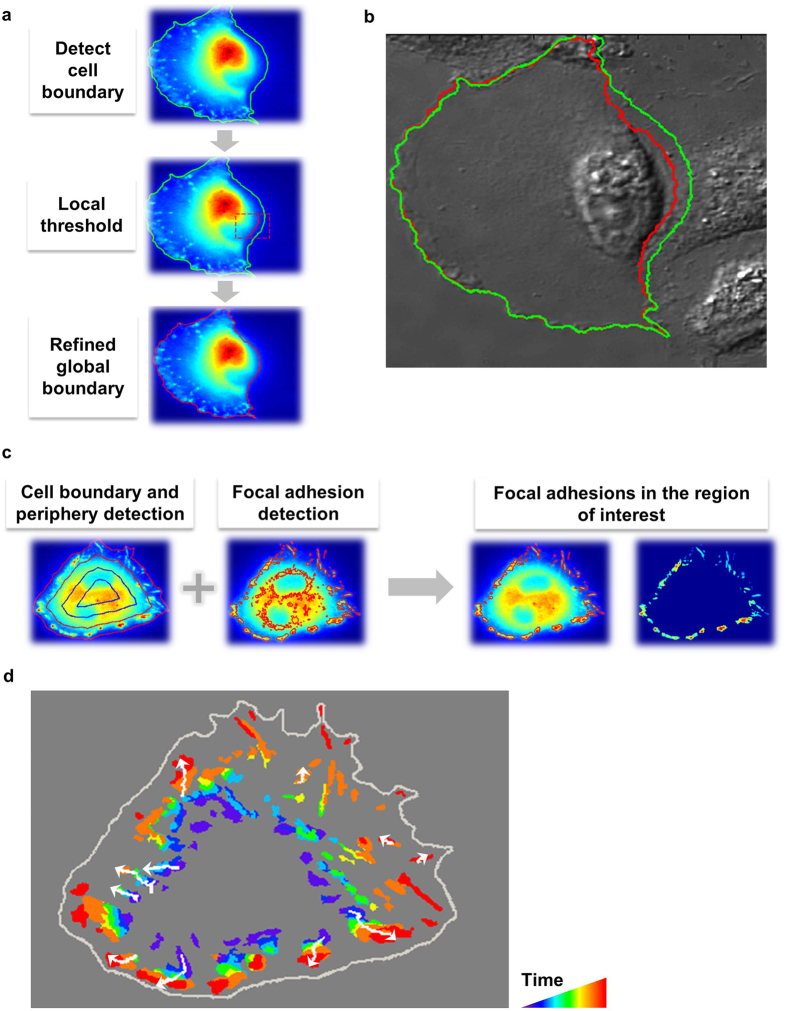
Cell and FA detection and tracking. **(a)** Detection of the cell boundary and subsequent refinement using local threshold values. **(b)** Comparison of the detected cell boundaries before (green) and after (red) refinement on a DIC image. **(c)** The detection schematics of FAs at the cell periphery. **(d)** The trajectories (white) of the well-tracked individual FAs that did not merge or split in a representative cell. The rainbow colors represent FAs at different time points in chronological order, with violet representing the FAs from the earliest frame and red the latest. Arrows indicate the direction of the FA translocation during the adhesion process.

The cell body was detected using the biosensor YPet images. Because of the marked change of YPet intensity profile during adhesion, Otsu’s method with an adaptive detection threshold was used to calculate the mask of the cell body^24^. The threshold was adapted continuously according to the decreasing YPet intensity resulted from cell spreading. In some cases, the YPet images of the cell did not have a uniform intensity profile around the cell boundary (Fig. 3a). As a result, segmentation based on a global threshold could not precisely detect parts of the cell boundaries (Fig. 3a). To address this problem, a two-step adaptive thresholding method was used to improve detection accuracy. First, we generated a basic cell boundary using a global threshold. Second, we detected the local thresholds and masks within a moving rectangle along the basic boundary by Otsu’s method (Fig. 3a). The local masks were then merged and smoothed to calculate the refined whole-cell boundary and mask (Fig. 3a). This two-step adaptive thresholding method improved the accuracy of cell boundary detection (Fig. 3b), and is robust for changing cell shapes and intensity profiles during adhesion.

After cell boundary detection, the FAs were subsequently detected using the modified water algorithm via high pass filter and thresholding^18^. The size of the high-pass filter was adjusted according to the cell size, allowing the accurate detection of dynamic FAs during adhesion (Fig. 3c). The cells were divided into layers based on the distance from each image pixel to the background region outside of the cell, allowing the determination of ECFP/FRET ratio and YPet intensity of FAs in the outermost layer composed mainly of lamellipodia regions (Fig. 3c).

Single-FA tracking is essential for the quantitative investigation of biosensor signals, FA dynamics, and their coordination at individual FA sites, since the average signals from a large number of cells or FA sites cannot reveal the precise coordination of molecular activations as different FAs generally undergo non-synchronized regulations. The single particle tracking algorithm developed by Jaqaman *et al*. was hence adopted here to track the detected FAs via optimization^38^ (Fig. 3d). We developed an interface program to overlay track numbers on the FA images, allowing a visual evaluation of the tracking accuracy. For the purpose of studying the molecular regulation of the assembly and disassembly of FAs, we focused on the FA population that grew or shrank in size, but did not split or merge. Hence, we used the these tracks of FAs for the quantification of the time courses of ECFP/FRET ratio and YPet intensity, as well as the subsequent analysis deciphering the coupling between the biochemical kinase activities and structural FA dynamics at individual FA sites.

### Src activation leads FAK activation following the initiation of FA assembly

**Figure 4:**
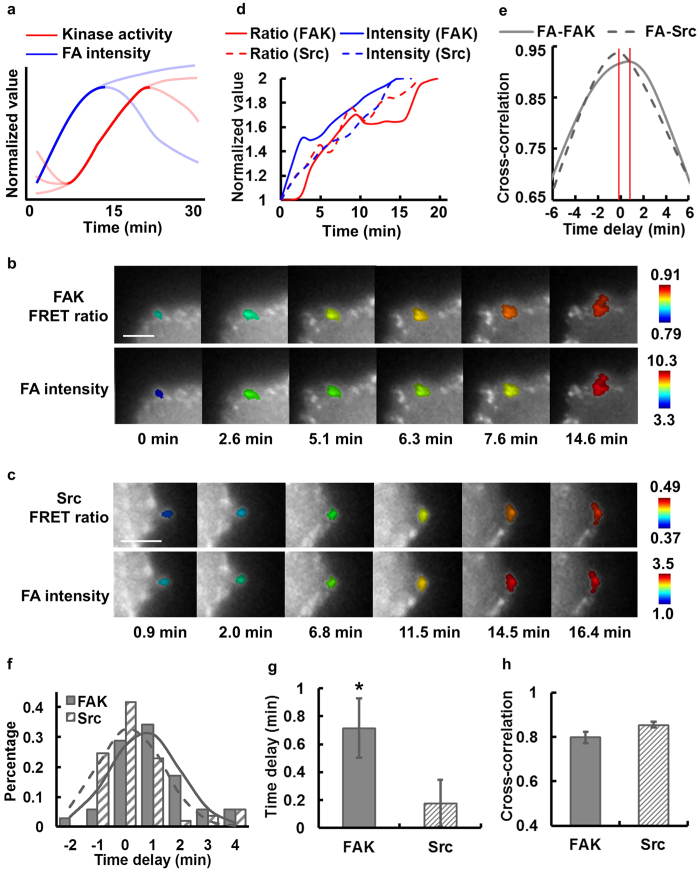
FA assembly leads the sequential activation of Src and FAK kinases. **(a)** The schematic of the typical kinase activity and total FA intensity kinetics at a single FA, with the rising phases shown in dark solid lines. The lines in light colors illustrate the variations in YPet intensity ECFP/FRET ratio trend among different individual FAs. In (**b,c**), the YPet intensity images of a representative FA color-coded by its ECFP/FRET ratio (top row) or by total FA YPet intensity (bottom row) in cells with either **(b)** FAT-FAK or **(c)** FAT-Src biosensors. **(d)** The time courses of the rising phases of ECFP/FRET ratio (red) and YPet intensity (blue) in representative FAs from different individual cells with either FAT-FAK (solid; images shown in panel b) or FAT-Src (dashed; images shown in panel c) biosensors. **(e)** The intensity-ratio cross-correlation functions calculated from the time courses in (b,c). **(f)** The histograms of the time delay, **(g)** the average time delays and **(h)** the cross-correlation peak values between the rising of YPet intensity and that of ECFP/FRET ratio in single FAs are compared between cells with the FAT-FAK biosensor (number of FAs: *n* = *35;* number of cells: *N* = *6*) and those with the FAT-Src biosensor (number of FAs: *n* = *53;* number of cells: *N* = *10*). * indicates statistically significant difference (*p* < *0.05*). Error bar: SEM. Scale bar: 5 μm.

Throughout the dynamic adhesion process of the MDA-MB-231 cells, some FAs continued to assemble and eventually matured, while others turned over or disassembled as depicted in the schematic plot (Fig. 4a). In contrast to the relatively smooth curves for FA intensity, the kinase activity shows more time-variance, with an overall rising phase accompanied by oscillatory patterns in time (Fig. 4a).

We first investigate the FA assembly phase. It has been reported that kinases are recruited to FA sites and activated to cause a positive feedback loop of phosphorylation, recruitment, and activation during FA assembly^4^,^39^,^40^. To investigate the sequential regulation between kinase activation and FA assembly, we first focused on the rising phase of ECFP/FRET ratio signals, which represents kinase activation, and that of YPet intensity, which represents FA assembly (Fig. 4b–d and Supplementary Videos S4-S5). No significant photobleaching was observed with our imaging setting (Fig. S3a) and evidenced in our previous publications^34^,^35^. Cross-correlation analysis^18^,^32^ was performed to evaluate the temporal phase difference between FA structural changes and kinase activation (Fig. 4e). In representative FAs, the cross-correlation function showed a positive time delay between the dynamics of YPet intensity and FAT-FAK ECFP/FRET ratio (Fig. 4b), indicating that FA assembly led FAK activation at this FA, while Src activation and FA assembly occurred nearly concurrently (Fig. 4b–e).

Cross-correlation analysis on multiple individual FAs showed a significant difference between the distributions of the time delays in FA-FAK and FA-Src correlations (Fig. 4f). These time delays were independent of the average YPet intensity of the corresponding FAs (Fig. S3b,c), suggesting that targeting our biosensors with Src or FAK substrates at FAs did not significantly perturb the endogenous molecular dynamics. Taken together, our results indicate that FA assembly occurred significantly earlier than FAK activation by 42.6 ± 12.6 sec, but not Src activation, with an FA-Src time lag of 10.8 ± 10.2 sec (Fig. 4g). The high peak values of the FA-FAK and FA-Src cross-correlations indicate a strong coupling between FA assembly and kinase activation (Fig. 4h). Although the FAT-Src and FAT-FAK activities were monitored in different cells, cross-correlation analysis allowed the quantitative evaluation of the Src and FAK activation sequence using FA signal as a common reference. Therefore, these results suggest that during FA assembly and growth, Src kinase is activated almost concurrently with FA assembly, which is on average 42.6 sec earlier than FAK activation at individual FA sites.

### The fate of individual FAs reflected by the kinase-FA coupling and regulated by the successive activations of FAK and Src

**Figure 5:**
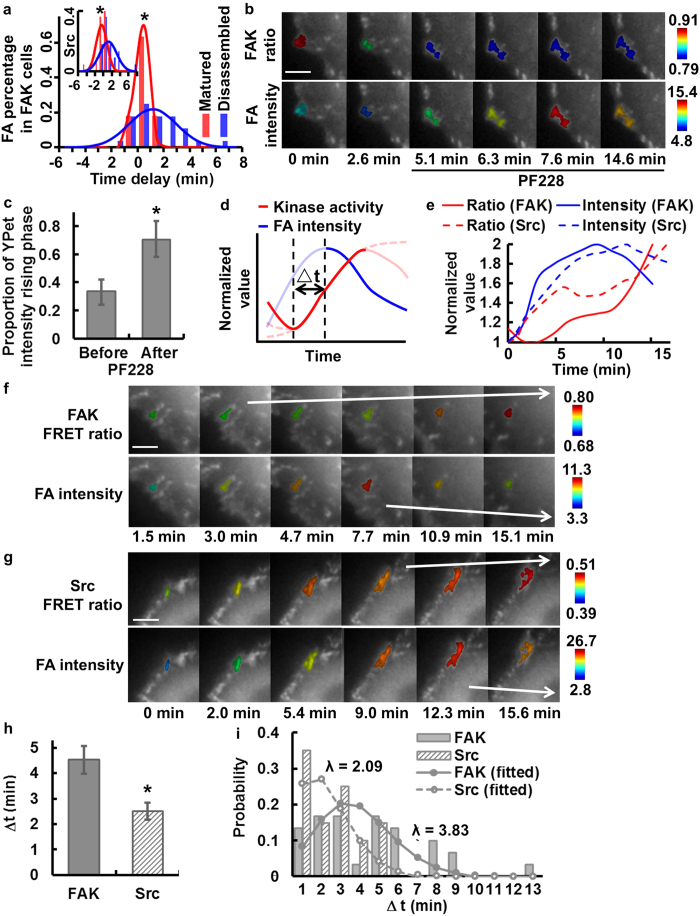
FAK activation leads Src activation and FA turnover sequentially. **(a)** The histograms of the single-FA time delays between the rising YPet intensity and ECFP/FRET ratio in different FA groups with the FAT-FAK biosensor or FAT-Src biosensor (insert). * indicates significant difference of the mean time delays between the matured and disassembled groups (*p* < *0.05*). **(b)** YPet intensity images of a cell before and after FAK inhibitor PF228 treatment, with a representative FA color-coded by its FAT-FAK ECFP/FRET ratio (top) or the total FA YPet intensity (bottom). **(c)** The portions of the YPet intensity rising phase in the periods before (34 ± 9%) and after (70 ± 13%) PF228 treatment in FAs (* indicates significant difference, *p* < *0.05;* number of FAs: *n* = *10;* number of cells: *N* = *1*). **(d)** The schematic of the typical kinase activity and total FA intensity kinetics at an individual FA highlighting the FA disassembly phase (solid blue) and its associated kinase activation phase (solid red). The time delay, △t, is defined as the difference between the starting points of FA disassembly (solid blue curve) and its associated kinase activation phase (the immediate preceding local minimum in ECFP/FRET ratio). The lines in light colors illustrate the variations in ECFP/FRET ratio and YPet intensity trends among individual FAs. **(e)** The time courses of the ECFP/FRET ratio and YPet intensity in two representative FAs, each in cells with FAT-FAK (solid) or FAT-Src (dashed) biosensors. YPet intensity images of cells with the representative FAs in panel (**e**) color-coded by the ECFP/FRET ratio (top) or the total FA YPet intensity (bottom) in cells with either **(f)** FAT-FAK or **(g)** FAT-Src biosensors. **(h)** Compare the time delay, △t (4.5 ± 0.5 min and 2.5 ± 0.3 min, respectively), calculated at individual FAs as described in (**d**) *: statistically significant difference by Kolmogorov-Smirnov test (*p* < *0.05, n1* = *30 in 6 cells, n2* = *20 in 10 cells*). **(i)** The fitted Poisson distribution of FAT-FAK △t (mean: *λ* = *3.66 min*) and that of FAT-Src △t (mean: *λ* = *1.33* *min*). Error bar: SEM. Scale bar: 5 μm.

To study the role of the kinases in determining the fates of FAs during disassembly, we separated the FA populations into matured (FA intensity increased or remained stable for 6 min or more) and disassembled (FA intensity peaked and then decreased) subgroups. Interestingly, the matured FAs in cells with the FAK biosensor had a sharp distribution of the time delay between FA assembly and kinase activation, with a mean value of 16.4 ± 9.4 sec (not significantly different from 0 sec, Fig. 5a), indicating nearly concurrent rises of FA intensity and kinase activity. In contrast, the disassembled FAs in cells with the FAK biosensor had a relatively dispersed distribution of the time delay between FA assembly and kinase activation with a significantly larger mean value of 69.6 ± 20.6 sec (Fig. 5a), suggesting that FA assembly led kinase activation by ~1.2 min in this group. Similar difference between the two groups of FAs also exists in cells with the Src biosensor, with the matured FAs having a sharp distribution of the time delay centered at −25.9 ± 16.4 sec (not significantly different from 0 sec), while the disassembled FAs having a significantly wider distribution centered at 69.5 ± 25.8 sec (Fig. 5a, insert). Statistical analysis shows a significant difference in the standard deviations of the time delays between the matured and disassembled FAs, which indicates distinct couplings between FA assembly and kinase activation in these two FA groups. It is of note that a wider distribution of FA-kinase time delay in the disassembled FAs can represent a less coordinated FA dynamics with its associated kinase activation and perhaps reflecting the dynamic instability of these FAs. This result suggests that, at the time of assembly, the fate of a FA to disassemble or to mature can be reflected by its FA-kinase coordination. FAs with tight coupling of FA-kinase have a high chance to mature and stabilize, while those with loose FA-kinase coupling mostly disassemble (Fig. 5a).

To probe further into the dynamic coupling between kinase activities and FA disassembly, we focused on the group of disassembled FAs. It has been documented that the activation of FAK/Src kinases at the FA site can lead to the disassembly of that FA^17^. To verify this notion in our system, we treated cells with the FAK inhibitor PF228 (1 μM) after the cells had stopped spreading. The inhibition of FAK activity reduced the FAK ECFP/FRET ratio and caused a more persistent FA growth than that in untreated cells (Fig. 5b and Supplementary Video S6). The persistency of FA growth was quantified from the ratio between the duration of the rising phase of YPet intensity and the total time course studied in individual FAs before or after inhibitor treatment. As shown in Fig. 5b,c, the persistency of FA growth significantly increased after PF228 treatment from 34 ± 9% to 70 ± 13%. In fact, the intensities of the majority of FAs kept increasing till the end of imaging, which was about 36 min after the addition of PF228. These results suggest that the inhibition of FAK leads to stabilization of FAs and that FAK activity plays an indispensable regulatory role in FA disassembly and turnover. This is also consistent with previous observations of a hindered FA disassembly in cells lacking FAK^17^ or SFKs^18^.

With the causal relationship between kinase activity and FA disassembly established, we proceeded to examine the temporal coordination between these two molecular events by investigating the time delay, △t, defined as the temporal difference between the starting point of the FA disassembly phase and its associated kinase activation phase (Fig. 5d). This approach avoids the difficulty of performing direct cross-correlation between kinase activation and FA disassembly engendered from multi-phasic oscillations of kinase activities during the lifetime of these disassembled FAs. FAK and Src ECFP/FRET ratio images and quantified time courses from representative FAs both showed an increase of kinase activities before the start of FA disassembly (Fig. 5e–g and Supplementary Videos S7-S8). The average △t in cells with the FAK biosensor was 4.5 ± 0.54 min, significantly larger than that of 2.5 ± 0.3 min in cells with the Src biosensor (Fig. 5h). By using the event of FA disassembly as a time reference, these results indicate that, at the single-FA level, the rising of biochemical FAK activity initiates ~2.0 min earlier than that of Src, which then lead to the structural FA disassembly in another ~2.5 min. Since the histogram of △t from individual FAs is similar to Poisson distribution, we fitted the histogram to the Poisson probability density function with least-squares approximation to obtain the parameter λ, the average time passed between the proceeding kinase activation wave and the start of FA disassembly. The fitted values of the time parameter λ confirmed that FAK activity rose ~2.0 min before Src activation in regulating FA disassembly (Fig. 5i). Since the distribution of △t fits the Poisson probability density function, this result suggests that the sequential activation of kinases may trigger FA disassembly via stochastic regulations, which occur in a time scale of minutes.

## Discussion

FAs are actively involved in mechanosensing, force transducing, and outside-in signaling during cell adhesion and expansion^9^,^20^. FAK and Src play key roles in the precise and fine regulation of the FA dynamics^15^,^17^. Our understanding on the regulatory functions of FAK and Src about FAs is mainly based on averaged signals from a large number of different cells/FAs at the global level, which can cause inaccuracies as individual FAs are generally not synchronized. Currently, there is a lack of imaging and analysis tools to quantitatively evaluate the temporal coordination among these different molecular events at individual FA sites. In this work, we engineered and characterized the FAT-Src and FAT-FAK FRET biosensors to precisely localize at individual FA sites and monitor the kinetic changes of Src and FAK activities, as well as the local FA structural change. With the aid of automated feature-detection and single particle tracking methods, we quantified the time sequences of FAK and Src kinase activation and FA intensity in a single FA throughout its dynamic lifespan during cell adhesion. By cross-correlation-based analysis^18^, we unraveled the kinetic coupling between the biochemical kinase activity and the biophysical structural dynamics at the single-FA level. Our results suggest that the FA complex assembly and its concurrent Src activation lead to a full-scale activation of FAK in ~40 sec. In guiding FA disassembly, however, the activation of FAK leads a wave of Src activation in minutes prior to the FA structural disassembly. Our analysis should thus provide a quantitative and precise understanding of the kinetic coupling among the molecular events within single FA complex during cell adhesion.

During the adhesion process, cells go through three major phases: the initial attachment (P0), rapid spreading (P1), and slow spreading (P2)^12^,^20^. Toward the end of P1 phase, the early FA proteins associated with integrin clusters, e.g., paxillin, talin, and FAK, recruit and activate Src kinase and formin to induce actin polymerization^15^,^41^. Subsequently, the force generated by actin flow and myosin contraction recruits vinculin and triggers FA maturation and cell spreading^16^,^42^,^43^. It is conceivable that the initial recruitment of kinases and the subsequent phosphorylation of substrate molecules within an FA complex facilitate the further recruitment of proteins for FA assembly. As the kinase activities accumulate, the FA proteins are hyper-phosphorylated to cause competing protein interactions within the complex, which may, together with the developed tension at the local site, lead to FA turnover or disassembly. As such, the fate of FA stabilization and turnover can be determined by the coordinated interplay of local biochemical kinase activities, biophysical force, and the molecular status of an FA. Our work highlights the roles played by FAK and Src kinases during this process, and provides a timeline of these sequential signaling events in the lifetime of an FA.

Our results revealed that FA assembly and the concurrent Src activation led FAK activation by ~43 sec in individual FAs. Although Src activation has been reported to precede FAK activation in cancer cell adhesion^44^, it is generally understood that FAK autophosphorylation at Y397 site was required for the recruitment of Src at the FA sites to fully activate FAK^45^,^46^,^47^. It is possible that the initial FAK autophosphorylation is relatively weak before the recruitment and activation of Src to turn on the full-scale activation of FAK, which then becomes detectable by the FAT-FAK biosensor^29^,^39^. A more sensitive FAK FRET biosensor may be needed to monitor this biphasic FAK activation, with the initial autophosphorylation and activation of FAK followed by a full-scale activation mediated by the Src recruitment and activation.

It is of note that there is a narrow distribution of cross-correlation time delays between the FA assembly and kinase activation in the matured FAs, suggesting a relatively coordinated coupling between FA structure and kinase regulations. In contrast, the time delays in disassembled FAs are significantly longer and dispersed across a wide range, suggesting a relatively stochastic decision-making process in the disassembled FAs. In fact, it appears that the fate of FA may have been determined during the assembly phase, with individual FAs having tight coupling between kinase activation and FA assembly inclining toward growth and maturation. It is possible that disassembled FAs are at locations where the subcellular microenvironment is less stable initially and hence require varied time periods to reach a deterministic process. In fact, oscillatory kinase activation waves can be observed in these disassembled FAs, possibly reflecting multiple rounds of adaptations and alterations of local biochemical processes.

**Figure 6:**
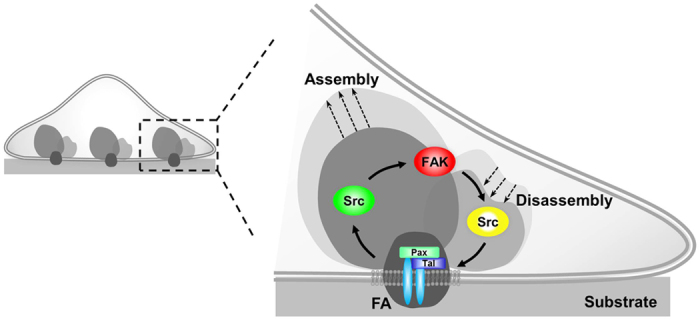
The proposed sequential signaling network of FAK and Src activations relative to the assembly and turnover of FAs at the single-FA level. During FA assembly, Src is activated earlier than FAK, while FAK activation in turn leads Src activation to trigger FA disassembly.

Different from the assembly phase, FA disassembly was demonstrated to be closely regulated by FAK and Src in our present and previous studies^17^,^18^. Our results suggest that FA disassembly follows the successive risings of FAK and Src activities within 4.5 minutes. As such, we propose a regulatory network within individual FA sites where Src activation leads a full-scale FAK activation after both Src and FAK are recruited during FA assembly. After reaching equilibrium, some FAs mature and become stabilized. Among the others that are destined to turnover, the full activation of FAK leads another wave of Src activation in triggering FA disassembly (Fig. 6). FAK may hence serve as a decision maker to switch FA from assembly toward disassembly phase in these turnover FAs, possibly via the accumulated FAK activity and its resulted hyper-phosphorylation of FA proteins aided by the lagging Src activation.

In summary, via the utilization of the newly designed FAT-FRET biosensors targeted at the subcellular individual FA sites, we have successfully monitored the active molecular events during cancer cell expansion upon adhesion. Single particle tracking and cross-correlation analysis provide powerful tools for the precise quantification of the temporal coordination among the biochemical activities of FAK and Src and the structural dynamics of the FA at the single-FA level. Unraveling the time sequence of FAK and Src activation dynamics at local FA sites should advance our in-depth understanding on how these crucial molecular events are coordinated to govern the cell adhesion that serves as a pivotal step for cancer cell metastasis to secondary sites. This understanding should also allow the design of new therapeutic approaches optimizing the inhibitor combinations and applications.

## Methods

### Focal adhesion (FA) targeting FRET biosensors

To engineer the FAT-FAK biosensor, a PCR product of the FAK-FAT domain with KpnI and EcoRI sites are first fused to the C-terminal of the cytosolic FAK biosensor (ECFP, SH2 domain, FAK substrate, and YPet) in pRSETB and the vector was replaced to pcDNA3.1 with BamHI/EcoRI sites for the mammalian expression^25^,^34^. To create the FAT-Src biosensor in pRSETB, the FAK biosensor part of pRSETB-FAT-FAK biosensor was replaced with a PCR product of the cytosolic Src biosensor (ECFP, SH2 domain, Src substrate, and YPet) containing BamHI/KpnI sites. For the mammalian expression, the pRSETBvector was further replaced by pcDNA3.1 with BamHI/EcoRI sites, generating FAT-Src biosensor in pcDNA3.1.

### Cell culture and reagents

Human breast cancer cells (MDA-MB-231) were cultured in DMEM supplemented with 10% fetal bovine serum (FBS), 2 mM L-glutamine, 100 unit/ml penicillin, 100 μg/ml streptomycin, and 1 mM sodium pyruvate (GIBCO BRL). Cells were maintained in a humidified 95% air, 5% CO_2_ incubator at 37 °C. Lipofectamine 2000 (Invitrogen) was used in the transfection of different DNA plasmids. The typical amounts of plasmids were 1.5–2.0 μg for single transfection of FAT-FAK or FAT-Src, and 1.0–1.5 μg each for co-transfection with mCherry-paxillin.

Cells expressing various exogenous proteins were starved in cell culture medium with 0.5% FBS for 36 hr before passing onto fibronectin-coated glass bottom dishes (Cell E&G) overnight prior to imaging. This step also served the purpose of synchronizing the status of cell cycle and reducing its effect on the variation of intracellular protein expression and molecular wiring. The dishes were incubated with FN solutions (20 μg/ml) at 37 °C for 4 hr before usage. For inhibitor experiments, cells with FAT-Src biosensors were pre-treated with the Src inhibitor PP1 (10 μM, BioMol) for 1 hour. A specific FAK inhibitor PF573228 (PF228, 1 μM, Pfizer) was added to cells with FAT-FAK biosensors during imaging^25^. PP1 and PF228 were dissolved in DMSO in stock solution (100 mM) and then further diluted in DMEM imaging medium before experiments.

### Image acquisition and analysis

Prior to microscopy imaging, the cells were transferred to Agarose gel dishes and suspended for 1 hr. The cells were then collected and transferred to glass bottom dishes for imaging of the adhesion process. Images were collected by a Zeiss Axiovert inverted microscope equipped with 1003 objective (1.4 NA) and a cooled charge-coupled device camera (Cascade 512 B; Photometrics) using the MetaFluor 6.2 software (Universal Imaging). The parameters of dichroic mirrors, excitation and emission filters for different fluorescence proteins were described previously^33^. In brief, the FAT-Src/FAK biosensors were excited at 420 ± 20 nm, and the emissions collected at 475 ± 40 nm or 535 ± 25 nm for ECFP or FRET images, respectively. The YPet images were obtained by excitation at 495 ± 10 nm and emission at 535 ± 25 nm. The mCherry-paxillin probe was excited at 560 ± 40 nm and the emission collected at 653 ± 95 nm for mCherry images. These excitation and emission settings were selected to allow optimal FRET efficiency and minimal bleed-through among FRET, YPet, and mCherry channels. Meanwhile, we minimized photobleach by reducing laser excitation duration and introducing neutral density filters to reduce excitation intensity, yet collect sufficient signals via a highly sensitive camera (Cascade II). As such, we will be able to obtain time-lapse images of single live cells without significant photobleach.

### Quantification and cross-correlation analysis

In signal processing, cross-correlation measures the similarity and time delay/difference between two signals. Cross-correlation analysis was applied in this study to reveal the dynamic coordination among FA dynamics (represented by the YPet intensity of the biosensors) and FAK, Src activities (represented by the ECFP/FRET ratio of the corresponding biosensors in cancer cell adhesion. We used the cross-correlation algorithm developed by Lu *et al*. to determine the time delay between FA assembly and kinase activations^18^. Instead of padding with zeros, the program extended the signals to a longer length using their end values and thus provided accurate results for slow and non-oscillatory signals, such as those observed during FA assembly and turnover. Both signals were normalized such that the maximum of the cross-correlation was one. The time-axis of the maximum of the cross-correlation function gives the time delay between the two signals analyzed. Since FAK and Src activities were not simultaneously monitored in the same cell, the FA dynamics served as a common reference in the temporal comparison between the two kinase activities.

### Statistical analysis

The Bonferroni multiple comparison test of means at 95% confidence interval was applied for the statistical analysis in Fig. 1e using a customized program based on the *multcompare* function in Matlab (The MathWorks, Natick, MA)^48^. Similarly, a paired multiple comparison test was performed for the statistical analysis in Fig. 2d using the same program. Two-tailed paired Student’s t-test (Type 1 TTEST, Excel) was used to compare the proportions of YPet intensity rising phase in the same FA before and after PF228 treatment in cells with the FAT-FAK biosensor in Fig. 5c. For the analysis in the assembly phase in Fig. 4f, the Kolmogorov-Smirnov test (kstest2, MATLAB) was utilized to compare the distributions of the time delays between FA assembly and kinase activation in cells with FAT-FAK and FAT-Src biosensors. To analyze the shapes of the cross-correlation time delay distributions in the matured and disassembled FAs, the randomized test (bootstrp, MATLAB) was used to compare the standard deviations of the distributions. For the rest of results, two-tailed two-sample Student’s t-test assuming different variances (Type 3 TTEST, Excel) was employed for the statistical analyses if not otherwise specified.

## Additional Information

**How to cite this article**: Wu, Y. *et al*. *In-situ* coupling between kinase activities and protein dynamics within single focal adhesions. *Sci. Rep.* **6**, 29377; doi: 10.1038/srep29377 (2016).

## Supplementary Material

Supplementary Information

Supplementary Video S1

Supplementary Video S2

Supplementary Video S3

Supplementary Video S4

Supplementary Video S5

Supplementary Video S6

Supplementary Video S7

Supplementary Video S8
