# Supplementary material for: In-situ coupling between kinase activities and protein dynamics within single focal adhesions
Source: Sci Rep. 2016 Jul 7;6:29377. doi: 10.1038/srep29377 (PMC4935953; doi:10.1038/srep29377)
Supplement: Supplementary Information [file srep29377-s1.pdf]

# *In-situ* coupling between kinase activities and protein dynamics within single focal adhesions

Yiqian Wu[1], Kaiwen Zhang[1], Jihye Seong[4], Jason Fan[2], Shu Chien[1,2], Yingxiao Wang[1,2*] and Shaoying Lu[1,2,3*]

[1]Department of Bioengineering, [2]Institute of Engineering in Medicine, [3]Center of Computational Mathematics, University of California, San Diego, La Jolla, CA 92093, USA, [4]Center for Neuro-Medicine, Brain Science Institute, Korea Institute of Science and Technology (KIST), Seoul, South Korea

[*] To whom correspondence should be addressed:

Shaoying Lu, Ph. D.

E-mail: kalu@eng.ucsd.edu

Yingxiao Wang, Ph. D.

E-mail:  yiw015@eng.ucsd.edu

## SUPPLEMENTARY FIGURE LEGENDS

**Supplementary Figure S1. The co-localization between FAT biosensors and paxillin.** **(a)** The intensity time courses of the YPet channel of the FAT-Src biosensor and mCherry-paxillin at the FA sties of the cell shown in Fig.1b. **(b)** The Pearson coefficients evaluating the co-localization between FAT biosensors and paxillin images of the cells in Figs. 1b (Src) and 2b (FAK). **(c)** The YPet intensity image of the FAT-FAK biosensor (left) is compared with the intensity image of paxillin-mCherry (middle) to generate the overlaid image (right) in one cell. **(d)** The intensity time course of the YPet channel of the FAT-FAK biosensor and mCherry-paxillin at the FA sites of the cell shown in (c).

**Supplementary Figure S2. The characterization of the FAT biosensors in MEFs.** **(a)** The ECFP/FRET ratio images of a representative MEF transfected with the FAT-Src biosensor during adhesion. **(b)** The ECFP/FRET ratio images of a representative MEF transfected with the FAT-FAK biosensor during adhesion. **(c)** The time courses of the ECFP/FRET ratio in the cell shown in (a) (black line with circles) and that in (b) (gray line with triangles). **(d)** The average ECFP/FRET ratio values before (0-3 min, 0.61 ± 0.048, white) and after (57-60 min, 0.81 ± 0.047, gray) the appearance of FAs in cells with the FAT-FAK biosensor ($p = 0.04, n = 3$), and those before (0-3 min, 0.40 ± 0.023, white) and after (57-60 min, 0.54 ± 0.039, gray) the appearance of FAs in cells with the FAT-Src biosensor ($p = 0.03, n = 4$). * indicates statistically significant difference. Scale bar: 10 μm.

**Supplementary Figure S3. The effects of photobleaching and biosensor targeting.** **(a)** The time course of average whole-cell YPet intensity in a stable adhering cell with the FAT-FAK biosensor. **(b)** The relationship between the temporal average biosensor YPet intensity and the cross-correlation time delay in the assembly phase of single FAs in a representative cell with FAT-FAK biosensor ($R^2 = -0.006$; number of FAs: $n = 13$) and **(c)** that in a representative cell with FAT-Src biosensor ($R^2 = -0.09$; number of FAs: $n = 9$).

**SUPPLEMENTARY VIDEO LEGENDS**

**Supplementary Video S1. The activation of Src kinase during cell adhesion and spreading.** The kinase activity of Src is visualized by the ECFP/FRET ratio of the transfected FAT-Src biosensor during the adhesion and spreading of the representative cell shown in Fig. 1b. The ECFP/FRET ratio turns from green to red, indicating an increase in Src activity.

**Supplementary Video S2. The dynamic activity of Src kinase during the adhesion and spreading processes of a PP1-pretreated cell.** The cell transfected with the FAT-Src biosensor and pretreated with PP1, corresponding to the one in Fig. 1c, develops fewer FAs with limited spreading and a lower ECFP/FRET ratio after touchdown as compared to the cell in Supplementary Video S1.

**Supplementary Video S3. The dynamic activity of FAK kinase during cell**

**adhesion and spreading.** The kinase activity of FAK is visualized by the ECFP/FRET ratio of the transfected FAT-FAK biosensor during the adhesion and spreading of the representative cell in Fig. 2b. The ECFP/FRET ratio turns from cool colors to warm colors as the cell spreads, and cools down after the addition of the FAK inhibitor PF228.

**Supplementary Video S4. FAK activity and FA dynamics at a single FA during its assembly.** Left: the YPet intensity images of the FAT-FAK biosensor were color-coded by the average FAK ECFP/FRET ratio within a representative FA at a corner of a cell. Right: the YPet intensity images were color-coded by the total YPet intensity within the same FA at the corner of the cell. A cold color indicates a low value while a hot color indicates a high one. This video corresponds to Fig. 4c.

**Supplementary Video S5. Src activity and FA dynamics at a single FA during its assembly.** Left: the YPet intensity images of the FAT-Src biosensor were color-coded by the average FAK ECFP/FRET ratio within a representative FA at a corner of a cell. Right: the YPet intensity images were color-coded by the total YPet intensity within the same FA at the corner of the cell. A cold color indicates a low value while a hot color indicates a high one. This video corresponds to Fig. 4d.

**Supplementary Video S6. FAK activity and FA dynamics at a single FA before and after PF228 treatment.** Left: the average ECFP/FRET ratio of the FAT-FAK

biosensor (color-coded) superimposed on the YPet intensity images of the biosensors within a representative FA in part of a cell. Right: the total YPet intensity (color-coded) superimposed on the YPet intensity images of a representative FA in part of a cell. A cold color indicates a low value while a hot color indicates a high one. This video corresponds to Fig. 5a.

**Supplementary Video S7. FAK activity and FA dynamics at a single FA over its lifespan.** Left: the average ECFP/FRET ratio of the FAT-FAK biosensor (color-coded) superimposed on the YPet intensity images of the biosensors within a representative FA in part of a cell. Right: the total YPet intensity (color-coded) superimposed on the YPet intensity images of a representative FA in part of a cell. A cold color indicates a low value while a hot color indicates a high one. This video corresponds to Fig. 5e.

**Supplementary Video S8. Src activity and FA dynamics at a single FA over its lifespan.** Left: the average ECFP/FRET ratio of the FAT-Src biosensor (color-coded) superimposed on the YPet intensity images of the biosensors within a representative FA in part of a cell. Right: the total YPet intensity (color-coded) superimposed on the YPet intensity images of a representative FA in part of a cell. A cold color indicates a low value while a hot color indicates a high one. This video corresponds to Fig. 5f.

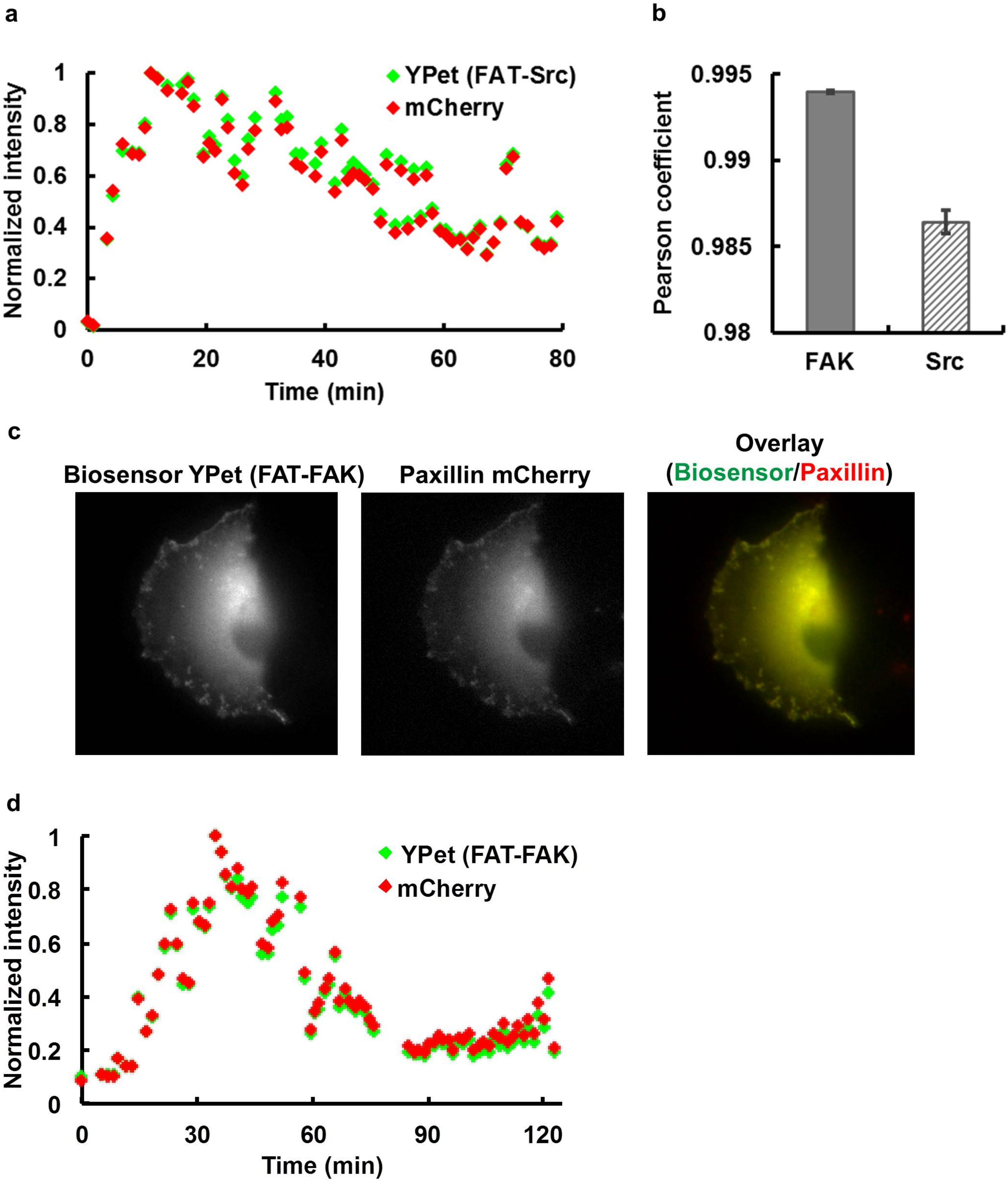


# Supplementary Figure S1

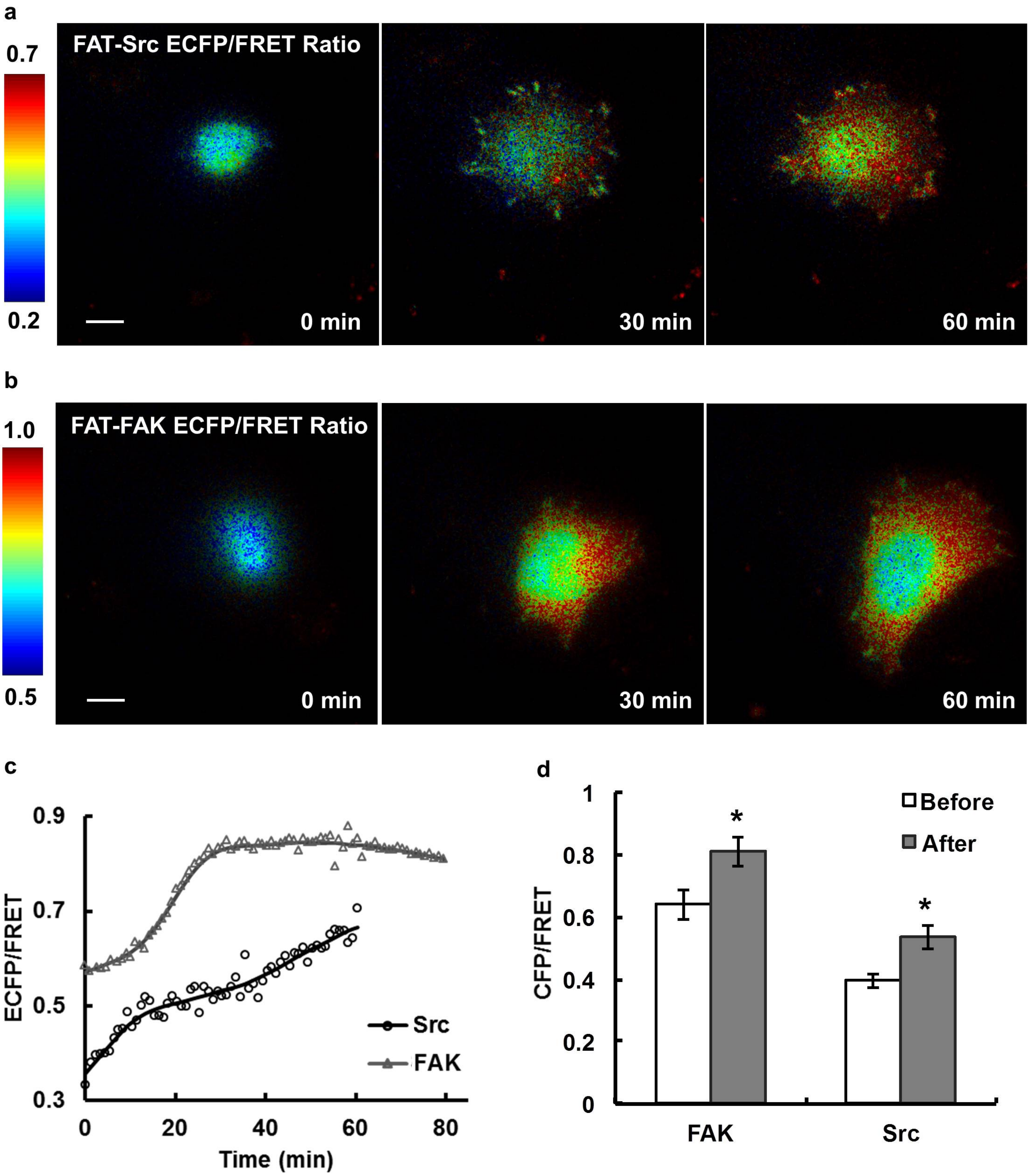


**Supplementary Figure S2**

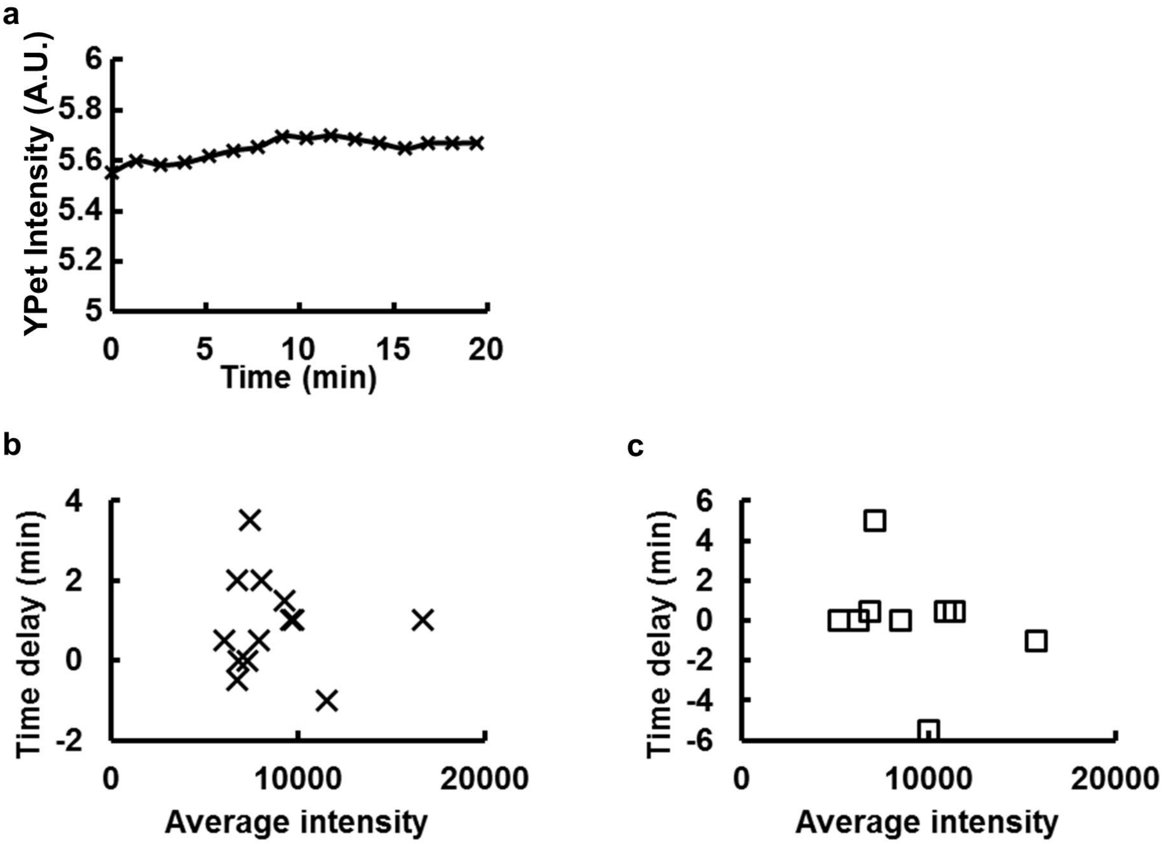


# Supplementary Figure S3
